# Neoadjuvant immunotherapy for colorectal cancer: Right regimens, right patients, right directions?

**DOI:** 10.3389/fimmu.2023.1120684

**Published:** 2023-03-06

**Authors:** Jiahao Zhu, Jie Lian, Benjie Xu, Xiangyi Pang, Shengjun Ji, Yutian Zhao, Haibo Lu

**Affiliations:** ^1^ Department of Outpatient Chemotherapy, Harbin Medical University Cancer Hospital, Harbin, Heilongjiang, China; ^2^ Department of Radiotherapy and Oncology, The Affiliated Suzhou Hospital of Nanjing Medical University, Gusu School, Nanjing Medical University, Suzhou, Jiangsu, China; ^3^ Department of Radiotherapy and Oncology, The Affiliated Hospital of Jiangnan University, Wuxi, Jiangsu, China

**Keywords:** colorectal cancer, immune checkpoint blockade, immunotherapy, microsatellite instability, neoadjuvant treatment

## Abstract

Neoadjuvant chemoradiotherapy (NACRT) or chemotherapy (NACT) followed by radical resection and then adjuvant therapy is considered the optimal treatment model for locally advanced colorectal cancer (LACRC). A recent total neoadjuvant therapy (TNT) strategy further improved the tumour regression rate preoperatively and reduced local-regional recurrence in locally advanced rectal cancer (LARC). However, distant metastasis was still high, and little overall survival benefit was obtained from these preoperative treatment models. According to mismatch repair protein expression, MSI-H/dMMR and non-MSI-H/pMMR statuses were defined in colorectal cancer (CRC) patients. Due to the special features of biologics in MSI-H/dMMR CRC patients, this subgroup of patients achieved little treatment efficacy from chemoradiotherapy but benefited from immune checkpoint inhibitors (ICIs). The KEYNOTE-177 trial observed favourable survival outcomes in metastatic CRC patients treated with one-line pembrolizumab with tolerable toxicity. Given the better systemic immune function, increased antigenic exposure, and improved long-term memory induction before surgery, neoadjuvant ICI (NAICI) treatment was proposed. The NICHE trial pioneered the use of NAICI treatment in LACRC, and recent reports from several phase II studies demonstrated satisfactory tumour downsizing in CRC. Preclinical rationales and preliminary early-phase human trials reveal the feasibility of NAICI therapy and the therapeutic efficacy provided by this treatment model. Better tumour regression before surgery also increases the possibility of organ preservation for low LARC. However, the optimal treatment strategy and effective biomarker identification for beneficiary selection remain unknown, and potential pitfalls exist, including tumour progression during neoadjuvant treatment due to drug resistance and surgery delay. Given these foundations and questions, further phase II or III trials with large samples need to be conducted to explore the right regimens for the right patients.

## Introduction

Colorectal cancer (CRC) remains the second leading cause of cancer-related mortality worldwide, with an estimated 93 5000 deaths in 2020 (including 576,858 deaths from colon cancer and 339,022 deaths from rectal cancer), accounting for 10% of all cancer types (1). Locally advanced CRC (LACRC) invading adjacent tissues and/or regional lymph nodes occur in 36% of initially diagnosed patients, and distant metastatic diseases occur in 22%. According to the statistics reported by the National Cancer Institute (USA), the 5-year overall survival (OS) rates for colon and rectal cancers are 64% and 67%, respectively (2).

Surgery combined with chemotherapy and radiotherapy remains the standard component of curative multimodal treatment approaches for LACRC. OS and disease-free survival (DFS) benefits from postoperative adjuvant chemotherapy were observed for LACRC patients in previous clinical trials (3–8). However, approximately 30% of CRC patients delayed or even refused adjuvant chemotherapy after curative resection because of postoperative complications and poor physical condition, and less than half of the eligible patients received a full course of chemotherapy, which reduced the therapeutic efficacy (9–11). To improve the possibility of completing chemotherapy prior to surgery, increase the drug concentration surrounding the tumour, eradicate potential micrometastases, reduce the risk of recurrence and distant metastasis, reduce the tumour bulk to achieve more complete resection, and assess the chemosensitivity of disease to predict the subsequent outcome, neoadjuvant chemotherapy (NACT) was proposed and applied to the treatment of CRC. The results of the FOxTROT trial and another randomized trial conducted in Germany raised the possibility of cure for CRC patients treated with neoadjuvant therapy, especially for those who achieved pathological complete response (pCR) (12, 13). However, an improved response may not translate into a survival benefit in locally advanced CRC (14–16). Then potential NACT regimen was explored. Oxaliplatin was added as the second cytotoxic agent during standard neoadjuvant chemoradiotherapy for locally advanced rectal cancer (LARC) patients in the PETACC-6 trial, but tumour response and survival benefits were not observed (17). Another phase II clinical study (PRODIGE 22) was terminated early due to the high grade of toxicities and unsatisfactory tumour regression (18).

The impressive tumour response of microsatellite instability high (MSI-H) or deficient mismatch repair (dMMR) metastatic CRC (mCRC) opened the era of immunotherapies for CRC in 2015 (19). The KEYNOTE-177 study suggested that immune checkpoint inhibitors (ICIs) have absolute advantages compared with traditional chemotherapy combined with targeted therapy in dMMR mCRC, which shows promising future opportunities for the use of ICIs for resectable CRC in the neoadjuvant setting (20). The NICHE trial reported favourable outcomes in which the pCR rate reached 60% for dMMR colon cancer patients who received a neoadjuvant immunotherapy regimen combining nivolumab (an anti-PD-1 inhibitor) with ipilimumab (an anti-CTLA-4 inhibitor) (21). In the VOLTAGE trial, 60% of dMMR LARC patients and 30% of proficient mismatch repair (pMMR) LARC patients who received NACRT followed by nivolumab achieved pCR (22). ICIs have a distinct toxicity profile and potential patterns of response compared with traditional cytotoxic chemotherapies (23, 24). Theoretically, more antigen surrounding the tumour could prime effective systemic immunity to eradicate potential metastatic disease in the neoadjuvant setting as opposed to the adjuvant use of this treatment postoperatively (25). In this review, we summarize the recent clinical developments of neoadjuvant ICI (NAICI) treatment in CRC and discuss the pitfalls and future directions of this approach.

## Management of locally advanced CRC

The primary aims of neoadjuvant treatment in LACRC are to achieve radical (R0) resection, decrease local recurrence and distant metastasis by pathological downstaging and eradicate occult micrometastatic disease with tolerable toxicity. Achieving a high rate of sphincter savings is also pursued in LARC. Breakthroughs of pCR or clinical complete response (cCR) achievement and organ preservation seem to have been made due to the application of radiotherapy and diverse neoadjuvant polychemotherapy patterns in LARC. Given the poor compliance of LARC patients, in which one-third of these patients refused to receive postoperative adjuvant chemotherapy and only 43% received 95% of the planned 5-fluorouracil (5-FU) dose in the standard neoadjuvant treatment model (EORTC 22921 study), adjuvant chemotherapy was proposed to be delivered before surgery, namely, total neoadjuvant therapy (TNT) treatment model (11). A significant downsize of the tumour before resection was observed in TNT models (26, 27), and the CAO/ARO/AIO-12 and OPRA trials further compared the therapeutic efficacy between the consolidation and induction treatment patterns in LARC (28, 29). Results of the two studies demonstrated that consolidation chemotherapy TNT model seems to have a better pCR rate, sphincter-saving rate and compliance than the induction model. Due to the higher rates of local-regional recurrence and relative fixed spatial position compared with colon cancer, radiotherapy is commonly applied in rectal cancer, especially for those mid-low rectal cancer. In terms of neoadjuvant radiotherapy in the TNT model, two radiotherapy methods are mainly applied in these trials: a short-course radiation scheme (5×5 Gy) and a long-course radiation scheme (25×2 Gy or 28×2 Gy). No difference in DFS was observed between the two radiotherapy model groups in the POLISH II and STELLAR trials (30, 31). The AVACROSS study reported an increase in the pCR rate to 36% at the cost of more serious surgery-associated complications when bevacizumab was added to induction chemotherapy (32). Poly ADP-ribose polymerase inhibitors (e.g., veliparib) and DNA protein kinase inhibitors (e.g., peposertib) are being evaluated in combination with NACRT or chemotherapy before surgery during TNT (22, 33–35). However, these studies are at an exploratory stage, and relative sequence studies deserve consideration. Overall, the TNT treatment model could improve the pCR rate for LARC compared with the traditional chemoradiotherapy model, which is consistent with the results obtained from three recent meta-analyses, while the survival benefits from TNT vary and deserve further investigation (36–38).

Limited literature has reported on neoadjuvant treatment in locally advanced colon cancer. Neoadjuvant chemotherapy with the OxMdG (fluorouracil and oxaliplatin) regimen achieved significant downstaging with acceptable gastrointestinal toxicity compared with standard postoperative chemotherapy in LACC, albeit with a low rate of pCR (2%) (12). The PRODIGE 22 study was stopped early due to a lack of efficacy in the FOLFOX (Folinic acid, fluorouracil, and oxaliplatin) combined with cetuximab arm (18). Another phase II trial analysed the therapeutic effects of panitumumab added to chemotherapy before surgery for LACC without KRAS, BRAF or PIK3CA mutations (39). The outcomes of this study showed that local recurrence was decreased to 26% and DFS was increased to 31% in patients with tumour downstaging, despite the high incidence of grade 3 skin rash. To date, there are no standard preoperative chemotherapy options for LACC, and an individual therapy model guided by multiplex gene testing or more accurate tumour and lymph node staging based on imaging before resection may provide new ideas for the treatment of this disease.

Tumour regression grading (TRG) systems are commonly used in gastrointestinal malignancies treated with neoadjuvant therapy, typically chemotherapy or radiochemotherapy. These categorizing methods assess the degree of tumour regressive changes by identifying fibrosis in relation to the residual tumour or evaluating the volume of the residual tumour in relation to the primary tumour lesions. TRGs could provide valuable prognostic information, and complete tumour regression has been confirmed to be closely associated with better survival and a lower recurrence rate in LARC (40). However, TRG cannot serve as a clinical research end-point due to the high numbers of versions of grading criteria and unclear conclusions on whether this classification system has advantages over the UICC/AJCC TNM staging systems. pCR is still used as a surrogate endpoint in neoadjuvant trials. NAICI trials have also adopted major pathological response (MPR) as the main endpoint, indicating that less than 10% of viable tumour tissues exist at primary sites after surgery in CRC and other cancers (41). Whether MPR could predict the survival of CRC patients who receive NAICI and serve as a surrogate for survival warrants further clarification and investigation.

## Rationale for NAICI in LACRC

### Advantages of NAICI in LACRC

**Figure 1:**
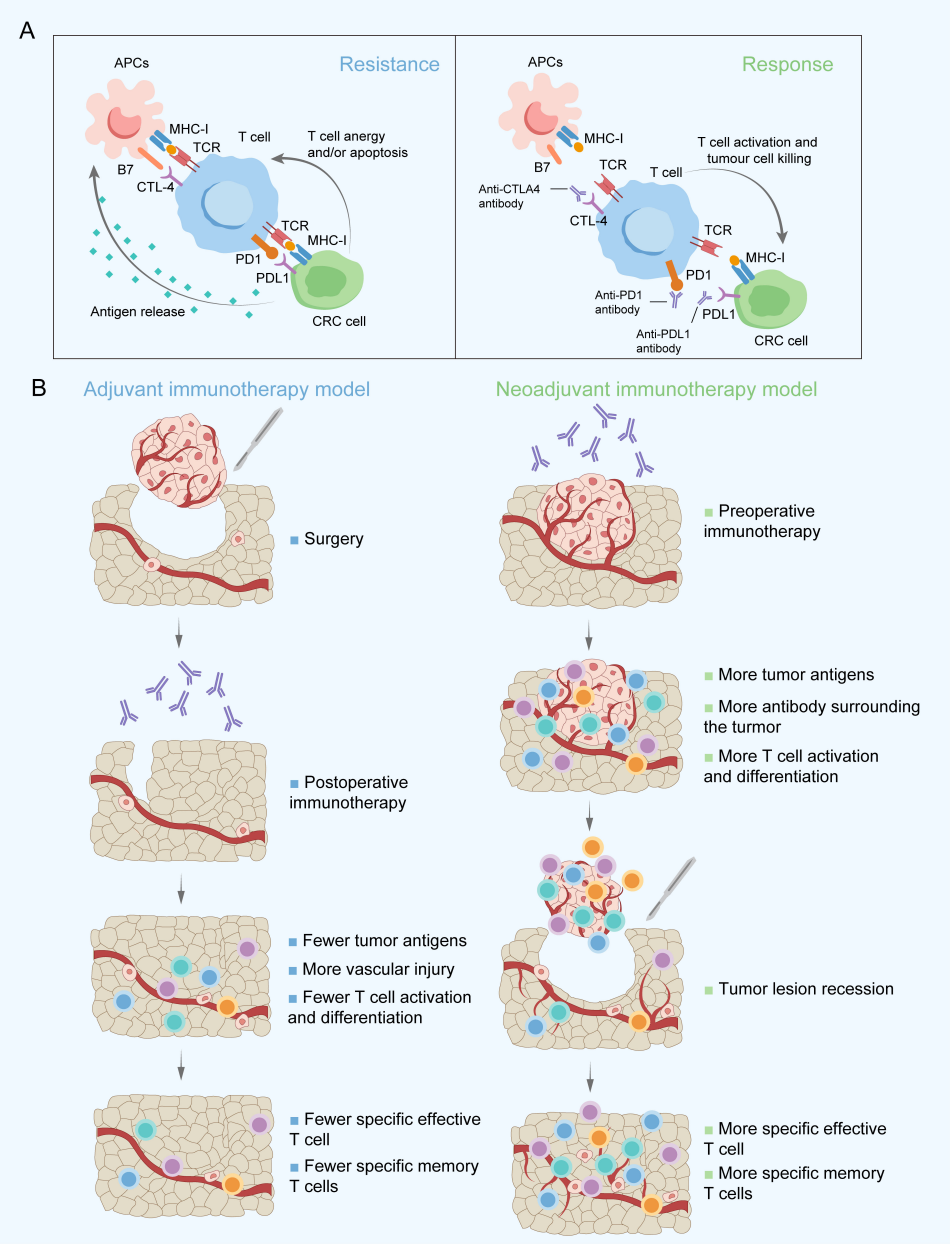
Main targets of immune checkpoint inhibitors applied in the clinic **(A)** and proposed rationale for immunotherapy administered postoperatively and preoperatively **(B)**. APCs, Antigen-presenting cells; CRC, Colorectal cancer; CTLA-4, Cytotoxic T-lymphocyte-associated antigen-4; MHC-I, Major histocompatibility complex class I; PD-1, Programmed death-1; PD-L1, Programmed death-ligand 1; TCR, T-cell receptor.

T-cell activation mainly depends on T-cell receptor (TCR) signalling participating in recognizing tumour antigens. Then, the immune response is activated, which is characterized by a large number of different T-cell clones (42). Tumour-infiltrating T cells, especially CD8+ T cells, have long been considered to have a close association with improving survival and reducing locoregional recurrence and distant metastasis by recognizing and killing specific tumour cells (43, 44). However, several immune checkpoint molecules, mainly cytotoxic T-lymphocyte-associated antigen-4 (CTLA-4), programmed death-1 (PD-1), and programmed death-ligand 1 (PD-L1), suppress T-cell-mediated antitumour immune responses in the tumour microenvironment (TME) (45–47). Meanwhile, high PD-L1 expression levels in tumour cells also contribute to the exhaustion and apoptosis of T cells (48) (**Figure 1A**). The clinical application of ICIs targeting CTLA-4 or PD-1/PD-L1 to reactivate the immune response against cancer have achieved favourable outcomes in advanced CRC, especially for patients with MSI-H/dMMR (20, 49). However, the benefits of survival are still not satisfactory from ICIs as adjuvant therapy, probably due to the significant decrease in tumour antigens and local-regional blood vessel and draining lymph node damage after surgery, which limit immune-mediated tumour cell killing and long-term tumour-specific immunological memory (**Figure 1B**). ICIs administered as neoadjuvant therapy instead of in an adjuvant setting may be considered to improve clinical outcomes. A greater expansion of T-cell clones in the peripheral blood because of a wider range of tumour antigen exposure and better survival were observed in preclinical mouse models when an ICIs were given preoperatively rather than postoperatively (25).

### Potential synergistic mechanism between NAICI and NACRT in LACRC

**Figure 2:**
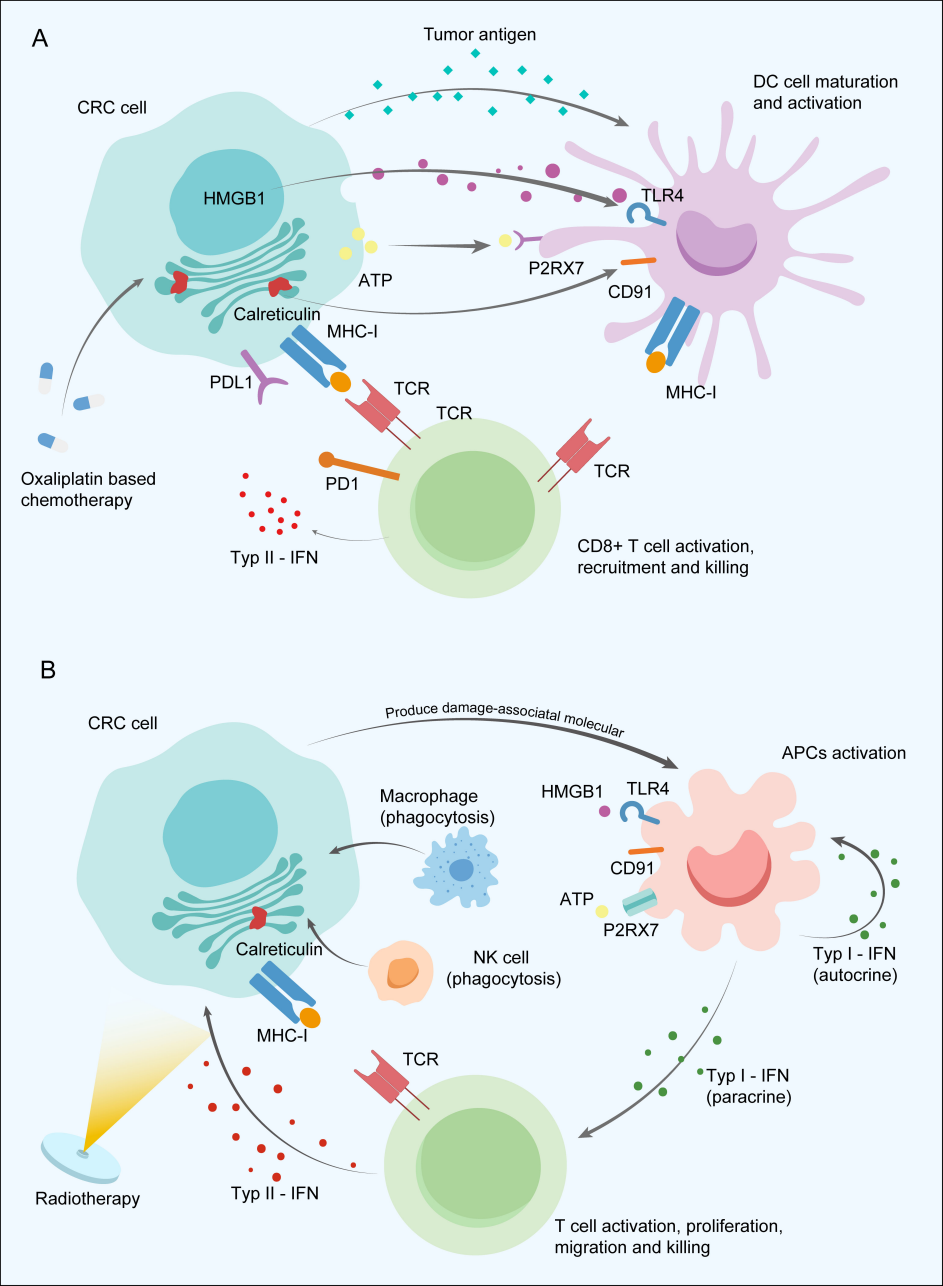
Potential synergistic mechanism between neoadjuvant immune checkpoint inhibitor and neoadjuvant chemotherapy **(A)** and radiotherapy **(B)** in colorectal cancer. APCs, Antigen-presenting cells; ATP, Adenosine triphosphate; CRC, Colorectal cancer; HMGB1, High-mobility group box 1 protein; IFNs, Type I interferons; MHC-I, Major histocompatibility complex class I; NK cells, Natural killer cells; P2RX7, Purinergic Receptor P2X 7 PD-1; Programmed death-1; PD-L1, Programmed death-ligand 1; TCR, T-cell receptor; TLR4, Toll Like Receptor 4.

Cytotoxic agents commonly used in the NACT of CRC mainly include oxaliplatin and 5-FU or its derivatives. Oxaliplatin could induce immunogenic cell death (ICD), a kind of regulated cell death, due to the ability to stimulate the pre-apoptotic release of calreticulin and to promote the release of high-mobility group box 1 protein (HMGB1) (50). ICD increases the intracellular antigen concentration and improves the availability of antigen-presenting cells (APCs), thus contributing to antigen presentation to cytotoxic T-lymphocytes and their priming. Oxaliplatin is also reported to enhance the antigen presentation capacity of tumour cells by increasing the surface expression of MHC class I, contributing to the more effective activation of T cells and ICI-based immunotherapy *in vivo* (51). Moreover, the combined use of oxaliplatin and 5-FU was observed to promote APC maturation in a colon cancer mouse model (52). In addition to regulating antigen presentation, the addition of oxaliplatin upregulated PD-L1 expression on tumour cells in a murine model of CRC and improved tumour control (53, 54). High expression of PD-L1 in tumour nests was found to have a close association with favourable outcomes after neoadjuvant chemotherapy and chemoradiotherapy (55, 56). Oxaliplatin was shown to increase the relative proportion of CD8+ T cells and promote the recruitment of cytotoxic T cells into the TME but selectively deplete B cells in BALB/c mice (57). Another study further demonstrated that chemotherapy with the FOLFOX regimen could regulate the tumour-infiltrating CD8+ T-cell exhaustion stage into effector functional status in CRC (58). 5-FU was found to activate cytotoxic T cells by contributing to the exhaustion of myeloid-derived suppressor cells *via* programmed cell death (59). The mechanisms of chemoimmunotherapy discovered in CRC provide promising prospects for use of NAICI together with oxaliplatin-based chemotherapy, warranting further exploration of the optimal therapeutic strategies to achieve a higher tumour response rate (**Figure 2A**).

Radiation mainly utilized in the neoadjuvant treatment of LARC reduces tumour size and improves local-regional disease control. Meanwhile, the immunostimulatory effects induced by radiotherapy and the potential possibility of improving antitumour immunity cannot be ignored. Tumour cell damage caused by irradiation releases a large amount of damage-associated molecular patterns, activating Toll-like receptors and other receptors on APCs, ultimately leading to phagocytosis *via* natural killer (NK) cells or macrophages and immunological signal priming. Type I interferons (IFNs) secreted by activated APCs contribute to APC maturation in the lymph node, antigen presentation function improvement through autocrine signalling, and CD8+ T-cell activation, proliferation, and migration into the TME *via* the paracrine pathway (60). Then, tumour-specific T cells secrete type II IFN to recruit T cells into tumour tissues and upregulate the expression of MHC-I on tumour cells with a feedback mechanism under radiation intervention (61) (**Figure 2B**). APCs also induce the generation of durable and transferrable memory responses mainly dependent on CD4+ helper T cells by presenting the tumour neoantigens to T cells (62). Overall, chemoradiotherapy was found to contribute to recruiting immune cells into the tumour site and upregulating immune checkpoint expression to promote to the synergistic enhancement of ICI therapy efficacy, which suggests a new and favourable approach to neoadjuvant therapeutic modalities for LACRC.

### Differences in the immune landscape between dMMR and pMMR in CRC

**Figure 3:**
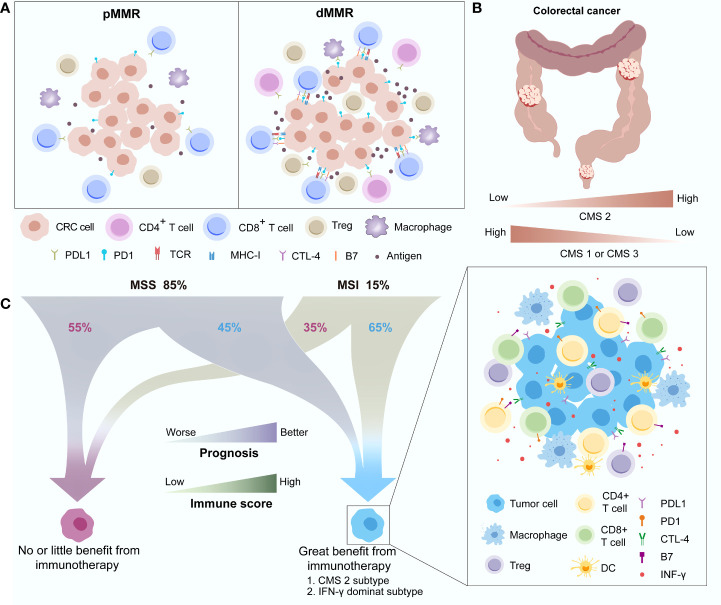
Differences in the immune landscape between pMMR and dMMR in CRC **(A)** and the distribution **(B)** and characteristics **(C)** of different immune subgroups. CMS, Consensus molecular subgroup; CRC, Colorectal cancer; CTLA-4, Cytotoxic T-lymphocyte-associated antigen-4; DC, Dendritic cell; dMMR, Deficient mismatch repair; IFN-γ, Interferon gamma; MSI, Microsatellite instability; MSS, Microsatellite stability; PD-1, Programmed death-1; PD-L1, Programmed death-ligand 1; pMMR, Proficient mismatch repair; TCR, T-cell receptor.

CRCs with MSI-H status account for approximately 15% of all cases of this disease. This special subtype of CRC marked with dMMR always has a high overall tumour mutation burden (TMB), which was reported to have a significant association with better ICIs treatment effects (63). High TMB means more neoantigen generation and thus recruits a large number of tumour-infiltrating immune cells, such as cytotoxic T cells, into the TME. The proportions of CD8+ T cells and CD4+ functional subsets were dramatically increased (64). To balance the immune response and protect normal host tissues, T-cell inhibitory ligands, such as CD80 and CD86 of the B7 family and PD-L1 as well as regulatory T cells (Tregs), are correspondingly upregulated in tumour cells to bind coinhibitory receptors, including PD-L1 and CTLA4 (19, 65) (**Figure 3A**). A consensus has been reached that early stage (not including stage IV) CRC and oesophageal adenocarcinoma with MSI-H status indeed have a higher rate of PD-L1 positive expression and high TMB at the same time compared with other tumours (66).

Interestingly, a recent study further divided MSI-H CRC into two major categories: MSI-H1 and MSI-H2 (67). Better OS was observed in the MSI-H2 group than in the MSI-H1 group (P = 0.042). Immune microenvironment differences between the two groups of CRC mainly involved the distribution of M2 macrophages and the expression of PD-L2. The anti-inflammatory and immune suppression functions of enriched M2 macrophages and T-cell inhibition functions of PD-L2 leading to immune regulatory disorder may contribute to the poor survival in the MSI-H1 subset. This finding is consistent with the fact that approximately 40%-60% of MSI-positive tumour patients cannot benefit from ICIs treatment (68). High immune heterogeneity also exists in pMMR or microsatellite stability (MSS) CRC. Dienstmann et al. proposed four consensus molecular subgroups (CMSs), including CMS1 (MSI immune), CMS2 (canonical), CMS3 (metabolic) and CMS4 (mesenchymal) based on the gene expression of CRC (69). CMS2, characterized by WNT and MYC activation, has better survival than the other CMSs, while CMS1, with the biological features of strong activation of immune evasion pathways, has worse survival after recurrence. CMS1 and CMS3 decreased while CMS2 increased as the tumour location moved distally, and CMS2 accounted for most rectal cancers (**Figure 3B**). Another study further investigated the relationship between the CMSs and the immune subtypes (C1-C6) established by Thorsson et al. (68, 70). This study discovered that the *IFN-γ–dominant* subtype (C2) has enhanced activation of immune system pathways and dominates in CMS1 (53%), while the *wound healing* subgroup (C1), mainly depending on metabolic pathways, occupies most of CMS2 (91%), CMS3 (77%) and CMS4 (77%). CMS2 and the *IFN-γ–dominant subtype* share a similar immune status, including strong immune activation (higher proportions of activated CD8+ T cells, activated CD4+ T cells, Tregs, dendritic cells, and M1/M2 polarization) as well as the upregulation of immune checkpoints (CTLA-4 and PD-1/PD-L1), which means that these subgroups of CRC patients may benefit more from ICIs treatment. Galon et al. observed that approximately 45% of MSS CRCs and 65% of MSI-H CRCs had a higher Immunoscore, an effective indicator for CRC relapse prediction based on the expression of CD3+ and CD8+ T cells surrounding the tumour, which could achieve a favourable antitumour response, while the rest with a low Immunoscore may be unable to benefit from ICIs treatment (71). Therefore, differences in the immune landscape between MSI-H and non-MSI-H CRC were observed, but high immune heterogeneity also existed in their respective subgroups (**Figure 3C**).

### Right regimens? NAICI strategies in CRC

Several large clinical studies have demonstrated survival advantages of MSI-H/dMMR mCRC treated with ICIs compared with traditional chemotherapy combined with or without ICIs therapy (19, 20, 49). The detection of MSI/MMR status for patients with an initial diagnosis of CRC recommended by the 2021 National Comprehensive Cancer Network (NCCN) guidelines makes the application of ICIs in early CRC possible (72, 73). In addition, the 2022 version of the NCCN guidelines also first recommend single PD-1 blockade (pembrolizumab) or a combination of anti-PD1 (nivolumab) and anti-CTL-4 (ipilimumab) as an alternative treatment for potentially resectable MSI-H/dMMR mCRC during the preoperative stage and MSI-H/dMMR cT4b colon cancer (74, 75). In terms of MSI-H/dMMR LACRC, several finished or ongoing clinical trials have explored the treatment efficacy of NAICI, and favourable outcomes have been achieved in some of these studies. MSS/pMMR CRC, accounting for approximately 85% of all CRC, benefit little from ICIs, which is a challenge for clinicians. Fortunately, the combination of ICIs and chemoradiotherapy has been widely applied in the neoadjuvant setting in recent clinical trials and has significantly improved tumour regression. Here, we summarize the clinical trials on the application of ICIs in the neoadjuvant treatment of CRC patients (**Table 1**).

**Table 1:** Reported studies involving neoadjuvant immunotherapy trials in CRC.

Identifier	Trial name	Enrollment	Location	Trial design	CR rate n(%)	MPR rate n(%)	Grade 3-4 AEs n(%)	Reference
NCT03854799	AVANA	101 (MSI-H:1, MSS:38, Rest unknown)	Rectal cancer	Single-arm, Stage II-III, Phase II, nCRT (50.4Gy+Cape) + Ave 10 mg/kg q2w×6 cycles → TME (8-10w after the end of nCRT)	pCR: 22 (23)	59 (61.5)	8% (non-immune related) and 4% (immune-related)	(76)
NCT03503630	Averectal	44	Rectal Cancer	Single-arm, Stage II-III, Phase II, SCRT(5x5Gy) → FOLFOX+ Ave 10 mg/kg q2w×6 cycles → TME (3-4w after last treatment)	pCR:15 (37.5)	27 (67.5)	Grade 3: 25 (58.1%) and Grade 4: 5 (11.6%)	(77)
NCT04911517	BFH-NCRTPD	pMMR: 20	Rectal Cancer	Single-arm, Stage II-III, Phase II, nCRT (50Gy+Cape) + Tisl 200mg q3w×3 cycles → Surgery → Adjuvant chemotherapy with CapeOx	pCR: 7 (58.3)	NR	3 (25) (enteritis, oral ulcer and hyperthyroidism)	(78)
NCT04165772		dMMR: 16	Rectal Cancer	Single-arm, Stage II-III, Phase II, Dost 500mg q3w×3 cycles → nCRT (50.4Gy+Cape) → TME	cCR: 12 (100)	NR	0	(79)
NCT03026140	NICHE	dMMR: 32 pMMR: 33	Colon cancer	Randomized, Stage I-III, Phase II, Ipi 1mg/kg×1 cycle + Nivo 3mg/kg q2w×2 cycle → Surgery (pMMR patients were randomly assigned to receive celecoxib from D1 until the day before surgery)	pCR: dMMR: 22 (68.8) pMMR: 4(13.3)	dMMR:31 (96.9) pMMR: 7 (23.3)	7 (10.7)	(80)
NCT03026140	NICHE-2	dMMR: 112	Colon cancer	Single-arm, Stage I-III, Phase II, Ipi 1mg/kg×1 cycle + Nivo 3mg/kg q2w×2 cycle → Surgery	pCR: 72 (67)	102 (95)	5 (4) (immune-related) and 15 (13) (surgery-related)	(81)
NCT04123925	NICOLE	dMMR: 3 pMMR:19	Colon cancer	Single-arm, Stage II-III, Phase II, Nivo 240 mg q2w×2 cycles → Surgery	NR	dMMR: 0 pMMR: 3 (15.8)	1 (4.5) (diarrhea)	(82)
NCT02921256	NRG-GI002	Pemb arm: 90 Control arm: 95	Rectal Cancer	Randomized, Stage II-III, Phase II, Pemb arm: FOLFOX×4m + nCRT(50.4Gy+Cape)+Pemb 200mg q3w×6 cycles → Surgery (8-12w after radiotherapy), Control arm: FOLFOX×4m + nCRT(50.4Gy+Cape) → Surgery (8-12w after radiotherapy)	pCR: Pemb arm: NR (31.9) Control arm: NR (29.4) cCR: Pemb arm: NR (13.9) Control arm: NR (13.6)	NR	Pemb arm: 48.2% (during nCRT) and 37.3% (after nCRT) Control arm: NR	(83)
NCT03102047	NSABP FR-2	MSS: 45	Rectal Cancer	Single-arm, Stage II-III, Phase II, nCRT → Durv 750mg q2w×4 cycles → Surgery (8-12w after radiotherapy)	pCR: 10 (22.2) cCR: 14 (31.1)	NR	NR	(84)
NCT04083365	PANDORA	60	Rectal Cancer	Single-arm, Stage II-III, Phase II, nCRT (50.4Gy+Cape) + Durv 1500mg q4w×3 cycles → Surgery (after 10-12w from neoadjuvant therapy)	pCR: 18 (32.7)	NR	4 (7.3) (diarrhea, skin toxicity, transaminase increase, lipase increase and pancolitis)	(85)
NCT04340401	PKUCH 04	dMMR: 27	Rectal Cancer	Single-arm, Stage II-III, Phase II, CapeOX + Cam 200mg q3w×3 cycles → Radiotherapy (45Gy) → CapeOX q3w×2 cycles → Surgery	pCR: 7 (33.3)	7 (33.3)	lymphopenia (24%), diarrhea (8%), and thrombocytopenia (4%)	(86)
NCT04518280	TORCH	Arm A: 35 Arm B: 32	Rectal Cancer	Randomized, Stage II-III, Phase II, Arm A: SCRT(5x5Gy) → CapeOX + Tori 240mg q3w×6 cycles → TME, Arm B: CapeOX + Tori 240mg q3w×2 cycles → SCRT(5x5Gy) → CapeOX + Tori 240mg q3w×4 cycles → TME	MSS: CR: 9 (81.8) pCR: 7 (77.8)	NR	Grade 3: 4 (36.4%) and Grade 4: 0	(87)
NCT02948348	VOLTAGE-A	MSS: 37 MSI-H: 5	Rectal cancer	Single-arm, Stage II-III, Phase Ib/II, nCRT (50.4Gy+Cape) + Nivo 240mg q2w×5 cycles → Surgery → Adjuvant chemotherapy with FOLFOX or XELOX	pCR: MSS: 11(30) MSI-H: 3(60)	MSS: 14(38) MSI-H: NR	2 (myasthenia and interstitial nephritis	(22)
NCT04231552		dMMR: 1 pMMR: 28 Unknown: 1	Rectal Cancer	Single-arm, Stage II-III, Phase II, SCRT(5x5Gy) → CapeOX + Cam 200mg q3w×2 cycles → Surgery (1w after last immunotherapy)	pCR: dMMR: 1 (100) pMMR: 12 (46.2)	NR	8 (26.7) (leukopenia, anemia and neutropenia)	(88)

Clinical trial details can be accessed at ClinicalTrials.gov database. AEs, Adverse effects; Ave, avelumab; Cam, Camrelizumab; CapeOX, capecitabine and oxaliplatin; Cap, capecitabine; cCR, Clinical complete response; CR, Complete response; D, day; dMMR, Deficient mismatch repair; Dost, Dostarlimab; Durv, Durvalumab; FOLFOX, folinic acid, fluorouracil and oxaliplatin; Ipi, Ipilimumab; MPR, Major pathologic response; MSI-H, Microsatellite instability-high; MSS, Microsatellite stability; m, Months; NCT, National Clinical Trial; Nivo, Nivolumab; nCRT, Neoadjuvant chemoradiotherapy; NR, Not report; Pemb, Pembrolizumab; pMMR, Proficient mismatch repair; pCR, Pathological complete response; SCRT, Short-course radiotherapy; Tisl, Tislelizumab; TME, Total mesorectal excision; W, Week; XELOX, Xeloda and oxaliplatin.

Due to the special clinicopathological and molecular biological features of MSI-H/dMMR CRC, the early-stage patients of these subgroups could not benefit from adjuvant chemotherapy. The phase II clinical trial NICHE (NCT03026140) adopted the combination of nivolumab and ipilimumab as the neoadjuvant regimen to explore the NAICI treatment efficacy (21). The short-term outcomes showed that all of the dMMR status patients achieved a pathological response with tolerable adverse effects. This study preliminarily proves that nonmetastatic colon cancer patients could benefit from NAICI and indicates that NAICI has become more acceptable and has a potentially wide application in CRC, especially for those with dMMR status. A recent updated oral report of this study showed that the pCR rate increased to 69%, and the NICHE-2 study with a single arm also showed a similar pCR rate (67%) (80, 81). The NICOLE study (NCT04123925) investigated the treatment efficacy of single ICIs before surgery in early-stage colon cancer without detecting MMR status (82). The results showed that all patients underwent radical resection without delay. The outcomes of the two studies suggest that NAICI treatment with a combination of anti-PD-1 and anti-CTL-4 agents seems to achieve better tumour regression than the application of a single anti-PD-1 agent. In terms of survival, only the NICHE study reported that no recurrence occurred in dMMR colon cancer patients with a median follow-up of 32 months, while 6% patients with pMMR status suffered from relapse with a median follow-up of 28 months. The long-term survival results of these clinical trials are expected.

In LARC, several studies have investigated whether adding ICIs before surgery to the standard neoadjuvant treatment model could further improve the tumour regression rate. In the VOLTAGE-A study (NCT02948348), nivolumab consolidation treatment were added between the period of standard NACRT and radical surgery in LARC patients with MSS or MSI-H status (22). Thirty percent (11/37) and 60% (3/5) of LARC patients with MSS status and MSI-H status, respectively, achieved pCR. Immune-related adverse effects were tolerable, and no treatment-related deaths occurred. This study pioneered the use of NAICIs in the traditional standard neoadjuvant treatment of LARC. The PANDORA study (NCT04083365) added durvalumab NAICI consolidation immunotherapy in the traditional neoadjuvant treatment strategy of LARC, and the pCR rate reached 32.7% (18/55) (85). However, the improved pCR rate of this clinical trial does not seem to exceed that of the TIMING trial, where the pCR rate increased to 38% in the MSS status subgroup (26). Then, NSABP FR-2 (NCT03102047) study added a NAICI treatment regimen with durvalumab between NACRT and surgery, and the outcomes of this clinical trial obtained a pCR of 22.2% and a cCR of 31.1% in MSS rectal cancer patients (84). BFH-NCRTPD (NCT04911517) reached a similar CR rate of 58.3% with a small sample. Tislelizumab were added during NACRT and before operation in this study (78). The TNT treatment model significantly improves the tumour regression rate with tolerable advances in LARC. Therefore, this kind of treatment model also serves as a good platform for NAICI treatment research in CRC. Similar to the different timings of chemotherapy intervention in TNT, various NAICI application models also exist in different clinical trials. In the PKUCH 04 study (NCT04340401),.7 (33.3%) achieved pCR among 21 patients who underwent surgery, and 4 obtained cCR or near cCR among those who chose the “watch and wait” strategy (86). No severe adverse events were observed. A study by Li et al. retrospectively analysed the pCR rate in pMMR/MSS LARC patients treated with a combination of TNT and ICIs (89). The results showed that 6 patients achieved pCR (30%) among the 20 patients who underwent surgery, and 3 patients achieved cCR among the 4 patients who refused to undergo surgery. Nine patients with pCR or cCR suffered from mild chemoradiotherapy and immune-related adverse effects, and no severe adverse effects were observed in any of these patients. Another retrospective study with a similar TNT treatment model without adding ICIs in the same patient group also reported a pCR rate of 29%, which suggests that this kind of ICIs intervention model seems to provide an additional therapeutic benefits (90). Two phase II studies, AVANA (NCT03854799) and NRG-GI002 (NCT02921256), investigated tumour regression when single ICIs were added concurrently with NACRT in LARC (76, 83). The TNT treatment model was applied in both clinical trials. In the AVANA study, a total of 23% (22/96) of patients achieved pCR, and 61.5% (59/96) of patients obtained an MPR. Another randomized controlled study, NRG-GI002, adopted a similar concurrent NAICI application model for stage II-III rectal cancer patients with high risk in the experimental group. However, no significant difference in the pCR rate was observed between the experimental group (31.9%) and the control group (29.4%) without pembrolizumab treatment.

All of the clinical trials in LARC above suggest that the pCR rate was not significantly improved when an NAICI was added to the TNT model. A potential reason is that long-term neoadjuvant radiotherapy damages the local immune system, limiting the immune response and reducing the additional efficacy of immunotherapy besides the prolonged surgical interval. Short-term neoadjuvant radiotherapy reduces impaired immune function and has noninferior treatment efficacy compared with long-term regimens in LARC according to the STELLAR and RAPIDO trials (16, 31). A study by Zhang et al. (NCT04231552) explored the treatment model of short-term neoadjuvant radiotherapy (5×5 Gy) followed by chemotherapy and ICI treatment in LARC patients (88). pCR rate was significantly increased to 46%. The interval between neoadjuvant treatment and surgery was greatly decreased, and no severe immune-related adverse events were observed. The AVERECTAL study also applied short-term neoadjuvant radiotherapy and increased the cycles of mFOLFOX6 and ICI before surgery in LARC patients (77). The pCR rate in this study significantly improved to 37.5% compared with 16% in the previous control group. A recent oral report of TORCH (NCT04518280), in which short-course neoadjuvant radiotherapy was administered, also showed an amazingly high CR rate (CR rate: 81.8%, 9/11; pCR rate: 77.8%, 7/9) in MSS LARC (87). The short-term neoadjuvant radiotherapy model seems to have a higher probability of achieving pCR in LARC when combined with an NAICI. The ongoing PRIME-PR (NCT04621370) study is directly comparing the tumour regression difference between short-term and long-term neoadjuvant radiotherapy in the TNT treatment model with NAICI in LARC (91). The TORCH (NCT04518280) study is further investigating the optimal intervention timing of NAICI and neoadjuvant chemotherapy administration for short-term neoadjuvant radiotherapy in the same population (92). Additionally, the REGINA study (NCT04503694) explored the therapeutic effect of the combination of nivolumab and regorafenib when administered before and after standard preoperative short-course radiation therapy in LARC (79). A recent study (NCT04165772) demonstrated that NAICI therapy with single dostarlimab, a PD-1 inhibitor, followed by radiotherapy without chemotherapy also achieved favourable tumour regression in dMMR stage II-III LARC (93). A total of 12 patients were enrolled, and all these patients achieved cCR. These completed or ongoing studies are currently in clinical phase II with limited examples, and the results of these studies are worthy of reflection and expectation. More large-size clinical trials with optimized treatment strategies are warranted. These currently recruiting studies are summarized in **Table 2**.

**Table 2:** Ongoing perspective studies involving neoadjuvant immunotherapy trials in resectable CRC.

Identifier	Trial name	Country	Estimated enrollment cases	Location	Trial design	Primary endpoint(s)	Estimated study Completion (year)	Reference
NCT04643041	BASKET	China	dMMR:47	Rectal cancer	Single-arm, NR, NR, Anti-PD-1 Antibody 200mg q3w×4 cycles → W&W	1-year DFS	2026	
NCT04621370	PRIME-RT	United Kingdom	48	Rectal cancer	Randomized, Stage II-III, Phase II, Arm A: Durv 1500mg×1 cycles → SCRT(5x5Gy) → FOLFOX×6 cycles+Durv 1500mg q4w → W&W or Surgery, Arm B: Durv 1500mg×1 cycles → Radiotherapy(50Gy+Cape)+Durv 1500mg q4w×1 cycles → FOLFOX×4 cycles+Durv 1500mg q4w → W&W or Surgery	CR	2025	(91)
NCT04503694	REGINA	Belgium	6	Rectal cancer	Single-arm, Stage II-III, Phase II, Nivo 240mg q2w×2 cycles+regorafenib 80mg d1-14 → SCRT(5x5Gy) → Nivo 240mg q2w×2 cycles+regorafenib 80mg d1-14 → TME → Adjuvant chemotherapy	pCR	2028	(79)
NCT03127007	R-IMMUNE	Belgium	54	Rectal cancer	Randomized, Stage II-III, Phase I/II, Arm A: nCRT (45-50Gy+5-FU) +Atez 1200mg q3w×4 cycle → Surgery, Arm B: nCRT (45-50Gy+5-FU) → Surgery	AEs, pCR	2023	
NCT04124601	CHINOREC	Austria	80	Rectal cancer	Randomized, Stage II-III, Phase II, Arm A: nCRT (50Gy+Cape) → Surgery Arm B: nCRT (50Gy+Cape) → Ipi 1mg/kg×1 cycle → Nivo 3mg/kg q2w×3 cycle → Surgery	AEs	2023	(94)
NCT04293419	DUREC	Spain	58	Rectal cancer	Single-arm, Stage II-III, Phase II, FOLFOX×4 cycles+Durv 1500mg q4w×3 cycles → nCRT (50.4Gy+Cape)+Durv 1500mg q4w×2 cycles → Durv 1500mg q4w×1 cycles → Surgery	pCR	2025	
NCT04017455	TARZAN	Netherlands	38	Rectal cancer	Single-arm, Resectable disease, Phase II, Radiotherapy → Beva 5mg/kg×1 cycle → Beva 5mg/kg+Atez 840mg×2 cycle → Atez 840mg×1 cycle → Surgery	cCR	2024	
NCT04906044	STARS-RC03	China	30	Rectal cancer	Single-arm, Stage II-III, Phase I, SCRT+chemotherapy+Tile → Surgery	AEs	2028	
NCT04130854	INNATE	American	58	Rectal cancer	Randomized, Stage II-III, Phase I/II, Arm A: SCRT(5x5Gy)+APX005M (an Anti-CD40 Agonist) 0.3mg/kg×1 cycle → APX005M 0.3mg/kg+FOLFOX×5 cycle → FOLFOX×1 cycle → TME, Arm B: SCRT(5x5Gy)→ FOLFOX×6 cycle → TME	pCR	2023	
NCT03299660		Australia	37	Rectal cancer	Single-arm, Stage II-III, Phase II, nCRT (50.4Gy+Cape or 5-FU) → Ave 10 mg/kg q2w×4 cycles → Surgery	pCR	2023	(95)
NCT04304209		China	195	Rectal cancer	Randomized, Stage II-III, Phase II/III, dMMR group: Sint 200mg q3w×4 cycles → Surgery or W&W → Adjuvant Sint ± CapeOX, pMMR group-1: nCRT (50Gy+CapeOX) + Sint 200mg q3w×4 cycles → Surgery or W&W → Adjuvant CapeOX×4 cycles, pMMR group-2: nCRT (50Gy+CapeOx) → Surgery or W&W → Adjuvant CapeOX×4 cycles	pCR	2026	(96)
NCT04109755	PEMREC	Switzerland	MSS: 25	Rectal cancer	Single-arm, Stage II-III, Phase II, SCRT(5x5Gy) + Pemb 200 mg q3w×4 cycle → Surgery	TRG	2028	(97)
NCT04411524		China	MSI-H: 50	Rectal cancer	Single-arm, Stage II-III, Phase II, Anti-PD-1 Antibody×2 cycles → nCRT (50Gy+Cape+Irinotecan) → Anti-PD-1 Antibody×3 cycles → TME → Adjuvant XELOX×6 cycles	pCR	2022	
NCT04411537		China	MSS: 50	Rectal cancer	Single-arm, Stage II-III, Phase II, Anti-PD-1 Antibody×2 cycles → nCRT (50Gy+Cape+Irinotecan) → Anti-PD-1 Antibody×3 cycles → TME → Adjuvant XELOX×6 cycles	pCR	2022	
NCT04443543		China	222	Rectal cancer	Non-Randomized, Stage II-III, Phase II, MSS group: XELIRI or FOLFIRINOX (cycles of chemotherapy depend on patient tumor responses) → Surgery or W&W MSI-H group: nCRT (50Gy+XELIRI or FOLFIRINOX) → Tisl 200mg×3 cycles → Surgery or W&W	cCR	2026	
NCT04357587		American	dMMR:10	Rectal cancer	Single-arm, Stage II-III, Phase I, nCRT (50Gy+Cape) + Pemb 200mg q3w×3 cycles → TME	AEs, Tolerability, Feasibility	2022	
NCT03921684		Israel	29	Rectal cancer	Single-arm, Stage II-III, Phase II, nCRT (50.4Gy+Cape) → FOLFOX+Nivo 240mg q2w×3 cycle → Surgery	pCR, AEs	2025	
NCT04558684		China	30	Rectal cancer	Single-arm, Stage II-III, Phase I/II, SCRT(5x5Gy) → CapeOX + Cam 200mg q3w×6 cycles → W&W or Surgery	cCR	2023	
NCT04663763		China	MSS: 32 MSI-H: 8	Rectal cancer	Single-arm, Stage II-III, Phase II, SCRT(5x5Gy) → CapeOX + Sint 200mg q3w×4 cycles → TME → Adjuvant CapeOX×4 cycles	pCR	2025	
NCT04636008		China	dMMR:20	Rectal cancer	Single-arm, Stage II-III, Phase Ib/II, SCRT(5x5Gy) → Sint 200mg q2w×3 cycles → Surgery	AEs	2022	
NCT03985891	JSFOL	China	40	Colon cancer	Randomized, Stage II-III, Phase I/II, Arm A: FOLFOX+JS001 (an Anti-PD-1 Antibody) 3mg/kg q2w×6 cycles → Surgery, Arm B: FOLFOX×6 cycles → Surgery	pCR, rCR, ORR	2026	
NCT05202314	NACSOC-02	China	20	Colon cancer	Single-arm, Resectable disease, Phase NR, Cam 200mg q3w×2 cycles+FOLFOX×3 cycles or CapeOx×2 cycles → Surgery	pCR	2026	
NCT04625803		China	64	Colon cancer	Single-arm, Stage II-III, Phase II, FOLFOX+Cam 200 mg q2w×5 cycles+Apatinib×2 m → Surgery → FOLFOX+Cam 200 mg q2w×7 cycles	TRG	2025	
NCT05231850		China	dMMR:70	Colon cancer	Single-arm, Stage II-III, Phase II, Tisl 200mg q3w until disease progression, unacceptable or withdrawal of consent	DFS	2027	
NCT03926338	PICC	China	dMMR: 100	Colorectal cancer	Randomized, Stage II-III, Phase I/II, Arm A: Tori 3mg/m^2^ q2w+Celecoxib×3m or 6m → Surgery Arm B: Tori 3mg/m^2^ q2w×3m or 6m → Surgery	pCR	2024	

Clinical trial details can be accessed at ClinicalTrials.gov database. 5-FU, 5-fluorouracil; AEs, Adverse effects; Atez, Atezolizumab; Ave, avelumab; Beva, bevacizumab; Cam, Camrelizumab; CapeOX, capecitabine and oxaliplatin; cCR, Cap, capecitabine; cCR, Clinical complete response; CR, Complete response; D, day; DFS, Disease free survival; dMMR, Deficient mismatch repair; Durv, Durvalumab; FOLFOX, folinic acid, fluorouracil and oxaliplatin; Ipi, Ipilimumab; m, Month; MSI-H, Microsatellite instability-high; MSS, Microsatellite stability; NCT, National Clinical Trial; Nivo, Nivolumab; nCRT, Neoadjuvant chemoradiotherapy; NR, Not report; ORR, Overall response rate; Pemb, Pembrolizumab; pMMR, Proficient mismatch repair; pCR, Pathological complete response; PD-1, programmed death-1; rCR, Radiographic complete response; SCRT, Short-course radiotherapy; Sint, Sintilimab; TME, Total mesorectal excision; Tori, Toripalimab; TRG, Tumor regression grade; W, Week; W&W, Watch & wait; XELIRI, Xeloda and irinotecan; XELOX, Xeloda and oxaliplatin; FOLFIRINOX, folinic acid, fluorouracil, irinotecan and oxaliplatin.

### Right patients? Potential biomarkers in NAICI

Currently, MSI/MMR status detection has been widely used for selecting potential patients who could benefit from immunotherapy in CRC and other solid tumours. A higher percentage of MSI-H/dMMR status subgroup patients benefited in terms of better tumour regression with NAICI treatment in the NICHE study and longer survival with first-line ICI treatment in the KEYNOTE-177 study (20, 21). MSI/MMR status seems to be an effective biomarker for predicting the response to immunotherapy, but only approximately 10% of localized CRCs and approximately 5% of mCRCs have MSI-H/dMMR status, and a few of MSS/pMMR CRC patients were also reported to benefit from ICI treatment (98, 99). Therefore, more precise and reliable predictors need to be identified. TMB, a biomarker measuring the somatic mutations per coding area of a tumour genome, may serve as a supplementary immunotherapy predictor in MSS/pMMR CRC (100). Similar to MSI-H/dMMR CRC, which have a 20 times higher mutation burden than non-MSI-H/pMMR CRC, a higher TMB is associated with a greater number of neoantigens produced and presented, enhancing immunogenicity and improving immunotherapeutic efficacy (101, 102). KEYNOTE-158 showed that the higher TMB subgroup shares a similar objective response rate regardless of MSI status in solid tumours (103). Then, two clinical trials preliminarily confirmed the treatment efficacy predictive value of TMB in CRC patients with MSS status who received immunotherapy (104, 105). TMB was detected in tumour tissue in the REGONIVO study, and a higher objective response rate (50% vs. 35.3%) and better median progression-free survival (12.5 vs. 7.9 months) were observed in the higher TMB group. Another CCTG CO.26 study also showed a better OS in CRC patients with TMB ≥ 28, in which plasma TMB was analysed with cfDNA in blood samples. Another tumor neoantigents related biomarker was POLE/POLD 1. Mutations in the POLE exonuclease domain disturbs the function of proofreading exonuclease activity required to replicate DNA with high fidelity and cause a high mutation burden in somatic cells (106). Most POLE-mutated CRCs have MSS or MSI-L statuses, while increased CD8+ T-cell infiltration, higher expression of T lymphocyte markers and effector cytokines, and upregulation of PD-1, PD-L1, and CTLA-4 could also be observed in these POLE-mutated CRCs (107, 108). Given that POLE-mutated CRCs have similar immune features to their MSI-H status counterparts, the application of immunotherapy deserves exploration, and therapeutic efficacy warrants investigation in POLE-mutated CRC.

Recently, B2M mutations were also found to be associated with clinical response in CRC patients treated with anti-PD-1 therapy. These mutations disturbed MHC class I antigen presentation, reduced T-cell activation, and contributed to immune escape. Different forms of B2M mutations showed the opposite prediction value in CRC. Primary B2M mutations were more frequently observed in MMR-deficient CRC, and complete B2M loss has a significant association with less recurrence and metastasis in CRC (109). However, acquired B2M mutations were reported to result in resistance to ICIs (65). Theoretically, PD-L1 expression could serve as a good clinical response predictor for tumours treated with anti-PD-1 therapy. The predictive value of PD-L1 expression has been confirmed in non-small cell lung cancer and gastric cancer, and PD-L1 detection with immunohistochemical staining has been widely used to guide the application of ICIs treatment in these tumours (110, 111). However, PD-L1 expression did not seem to be associated with prognosis in CRC patients (19, 49). Both of the two genes participate in the stimulation of immune effector cells, and the unusual prognostic role of the two genes in CRC treated with anti-PD1 therapy warrants further elucidation of the potential mechanism.

A previous study demonstrated that a higher density of tumour-infiltrating lymphocytes (TILs), especially CD8-positive T cells, surrounding CRC tumours was significantly associated with a better ICIs treatment response (112). In the NICHE study, CD8-positive T-cell infiltration (TCI) and CD8-positive PD-1-positive TCI were found to be higher in dMMR status colon cancer than that in its pMMR counterpart. Further molecular and immunophenotypic analyses revealed that CD8-positive PD-1-positive TCI could predict the ICIs treatment response in pMMR colon cancer and was considered an effective predictor for ICIs therapy (21). In addition to CD8-positive TCI, the density of CD3-positive T cells was also found to be a more favourable prognostic biomarker in CRC than common histopathological prognostic factors (43). The Immunoscore was proposed as a standardized method to assess the density of both intra- and extratumoral CD3-positive T cells and CD8-positive T cells in colon cancer (44). Its favourable prognostic predictive value has been validated in 3539 patients with stage I-III colon cancer from 14 expert centres in 13 countries. Further validation of the Immunoscore in CRC is ongoing in a multicentre clinical trial (NCT01688232). Another phase II study (NCT04262687) evaluated the treatment efficacy of a chemotherapy regimen of XELOX (xeloda and oxaliplatin) and bevacizumab combined with pembrolizumab in unresectable mCRC patients with an MSS status and a higher level of immune infiltration. More precise and reliable predictive biomarkers need to be identified to select potential patients who could benefit from ICIs treatment.

### Right directions? Pitfalls and promise of NAICI in CRC

The current outcomes reported from completed or ongoing clinical trials have revealed that tumour regression benefits from the application of NAICI in locally advanced CRC, but the optimal timing and strategies of immunotherapy remain unknown, and beneficiary identification needs further exploration. Additionally, several concerns regarding ICIs treatment should not be ignored. One concern is that early progression of disease during the period of neoadjuvant treatment due to drug resistance may result in CRC patients losing their best surgical chances. As shown in the results of the PANDORA study, local and/or metastatic tumour progression occurred in 3 patients before surgery (85). The potential mechanisms of ICIs treatment resistance are complicated. Grasso et al. investigated the potential immune escape mechanisms in MSI-H/dMMR CRC patients (113). They found that genetic alterations influenced the multiple steps of immune activation. Changes in the WNT signalling pathway impaired immune recognition. Genetic alterations, including a biallelic loss of β2 microglobulin (β2M), an MHC class I component, and single-copy loss events in HLA molecules lead to antigen presentation defects. Alterations in immune response–related genes involved in T-cell reactions, B-cell differentiation and NK cell activity contributed to weakened immune function. These potential immune pathway changes may cause immune tolerance and intrinsic resistance to ICIs treatment. Among these various mechanisms, *IFN-γ* signalling was found to play a key role in immunotherapy resistance. *IFN-γ* signalling initiated by T cells could induce PD-L1 expression through JAK-STAT signalling and interfere with the combination of CTLA-4 and the costimulatory molecule B7. Melanoma patients with JAK-STAT signalling-related gene mutations experienced tumour recurrence after responding to anti-PD-1 therapy (114). *IFN-γ* signalling-related gene mutations caused a nonresponse to anti-CTLA-4 treatment (115).

Another concern is the immune-related adverse events (irAEs) that occur during NAICI treatment in CRC. Severe irAEs prolong the interval between neoadjuvant treatment and surgery, increase the possibility of potential distant metastasis, and even cause mortality. Theoretically, patients who receive NAICI treatment may be more vulnerable to severe irAEs due to the more functional immune system compared with late cancer. Colitis is the most common irAE in patients treated with ipilimumab monotherapy, while hypothyroidism, rash, and diarrhoea are more commonly observed in patients treated with nivolumab and pembrolizumab (116). A retrospective study by Han et al. reported that 58.3% of dMMR CRC patients were treated with anti-PD1 neoadjuvant monotherapy, including pembrolizumab or nivolumab, and 16.7% of these patients experienced grade 3-4 irAEs (117). A combination of anti-PD1 and anti-CTL-4 immune drugs was applied in the NICHE study; 13% of patients suffered from grade 3-4 immune treatment-related toxicity, and none of the patients delayed radical resection (21). In a study by Zhang et al., camrelizumab (anti-PD-1) and chemotherapy were applied after short-course radiotherapy, and no grade 3-4 irAEs were observed (77). Outcomes of these small sample clinical trials showed that severe irAEs, mainly colitis, that occurred during NAICI treatment in CRC were uncommon and manageable and did not affect the timing of surgery. However, the number of patients enrolled in these studies was limited, and potential irAEs still warrant full and serious consideration in daily practice.

Except for these pitfalls, the application of NAICI therapy further improves the possibility of organ preservation (OP) in LARC patients. For patients who achieve cCR after neoadjuvant treatment, whether rectal resection should be performed is controversial. Harba-Gamal et al. first proposed the watch and wait (W&W) strategy in 2004 (13). They advised carrying out close follow-up rather than performing total mesorectal excision for these cCR LARC patients. This strategy preserves functional issues and avoids the complications of surgery, including anal dysfunction and sexual dysfunction, which improves the quality of life of these patients. A secondary analysis of the OPRA clinical trial analysed the differences in survival and OP rates among LARC patients who achieved cCR, near complete response (nCR), or incomplete clinical response (iCR) after TNT treatment (118). cCR and nCR patients were assigned to the W&W strategy, while iCR patients were recommended to undergo total mesorectal excision. The outcome of this analysis demonstrated that the 3-year OP rates in cCR and nCR patients were 79% and 52%, respectively, and the 3-year DFS in the iCR group was lower than that in the other two groups. However, no significant difference in the 3-year OS was observed among these three subgroups. They recommended the W&W strategy as a feasible treatment model for LARC patients who achieved cCR or nCR after neoadjuvant therapy. Long-lasting benefits have been observed in several cancers if favourable immunotherapy efficacy was obtained from initial immune treatment (80, 119, 120). Maintenance immunotherapy may further improve the long-term OP and DFS rates in LARC patients who achieved cCR or nCR after NAICI therapy, especially for those in the MSI-H/dMMR subgroup. Further large sample size preclinical validation studies are warranted.

## Future prospects

The optimal intervention timing of immunotherapy, ideal duration of ICIs, appropriate dose of immune drugs, and combination strategy of ICIs and cytotoxic agents before surgery are unknown now. A large number of early exploratory clinical trials are ongoing. The treatment model of short-course radiotherapy followed by 4-5 cycles of anti-PD1 therapy combined with fluorouracil- or its derivative-based chemotherapy before surgery in CRC seems to be more ideal according to reports to date. Screening of beneficiaries of immunotherapy remains based on the MSI/MMR status, while some MSI-H/dMMR CRC patients could not benefit from ICIs treatment, and some MSS/pMMR patients could achieve a good clinical response from immunotherapy. Therefore, response-associated genetic signatures covering neoantigen load, immune system response to tumour or antigen load and any defects in reaction initiation should be established (42). Pathological alterations, TME changes and potential mechanisms of ICIs resistance could be deeply investigated with residual viable tumour tissues obtained after NAICI therapy by surgical resection. Single-cell analysis of these tumour specimens could reveal immune evasion mechanisms from multiple dimensions. Findings from such studies may convert immunologically “cold” tumours to “hot” tumours and lead to the development of new treatment combinations to improve ICIs treatment efficacy. Robust biomarker identification will also reduce the risk of patients suffering from severe irAEs. Additionally, a higher level of tumour antigen-specific circulating T cells induced by NAICI could develop a long-lasting effector-memory T-cell pool (25, 121, 122). This finding may provide a potential survival benefit for patients treated with NAICI who achieved cCR or nCR if maintenance immunotherapy was administered without surgery.

## Conclusion

The application of neoadjuvant therapy in CRC downsizes the tumours preoperatively and improves local and systemic control of the disease, but no significant OS benefit was observed in previous clinical trials (36–38). Favourable outcomes were obtained in dMMR mCRC patients from treatment with pembrolizumab monotherapy as first-line therapy. The administration of ICIs was pioneered in LACRC in the neoadjuvant setting, which yielded impressive results especially in MSI-H/dMMR patients (20). Possible reason is due to the enhanced T-cell activation when a more functional immune system encountered more antigenic exposure before operation. However, optimum NAICI treatment regimen establishment and effective biomarker identification for beneficiary screening need further exploration. In summary, greater cooperation between clinicians and tumour immunologists contributes to a deeper understanding of the mechanisms of action of ICIs and to the development of robust theoretical foundations for the improvement of ICIs treatment efficacy in cancers.

## Author contributions

JZ, SJ, YZ and HL conceived and designed this study. JL, BX, and XP drew figures and tables. JL, BX, and XP collected and updated clinical trial data. JZ wrote the manuscript and HL revised the manuscript. All authors contributed to the article and approved the submitted version.

## Glossary

**Table:**

5-FU	5-fluorouracil
AJCC	American Joint Committee on Cancer
APCs	Antigen-presenting cells
B2M	Beta-2-Microglobulin
BRAF	V-raf murine sarcoma viral oncogene homolog B1
CapeOX	Capecitabine and oxaliplatin
cCR	Clinical complete response
CCR5	C-C chemokine receptor type 5
CD3+	Cluster of differentiation 3 positive
CD4+	Cluster of differentiation 4 positive
CD8+	Cluster of differentiation 8 positive
cfDNA	Circluting-tumor DNA
CMSs	Consensus molecular subgroups
CRC	Colorectal cancer
CTLA-4	Cytotoxic T-lymphocyte-associated antigen-4
CXCR3	Cxc chemokine receptor 3
DFS	Disease free survival
dMMR	Deficient mismatch repair
FOLFIRINOX	Fluorouracil
irinotecan	and oxaliplatin
FOLFOX	Folinic acid, fluorouracil, and oxaliplatin
HMGB1	High-mobility group box 1 protein
ICD	Induce immunogenic cell death
ICIs	Immune checkpoint inhibitors
iCR	Incomplete clinical response
IFNs	Type I interferons
IFN-γ	Interferon gamma
irAEs	Immune-related adverse events
JAK-STAT	Janus kinase-Signal transducer and activator of transcription
KRAS	Kirsten rats arcomaviral oncogene homolog
LACRC	Locally advanced colorectal cancer
LARC	Locally advanced rectal cancer
mCRC	metastatic colorectal cancer
MHC-I	Major histocompatibility complex class I
MPR	Major pathological response
MSI	Microsatellite instability
MSI-H	Microsatellite instability high
MSI-L	Microsatellite instability low
MSS	Microsatellite stability
NACT	Neoadjuvant chemotherapy
NACRT	neoadjuvant chemoradiotherapy
NAICI	Neoadjuvant Immune checkpoint inhibitor
NCCN	National Comprehensive Cancer Network
nCR	Near complete response
NK cells	Natural killer cells
OP	Organ preservation
OS	Overall survival
OxMdG	Fluorouracil and oxaliplatin
pCR	Pathological complete response
PD-1	Programmed death-1
PD-L1	Programmed death-ligand 1
PI3KCA	Phosphatidylinositol-4,5-bisphosphate 3-kinase, catalytic subunit alpha
pMMR	Proficient mismatch repair
POLD 1	polymerase delta 1
POLE	Polymerase epsilon
TCI	T-cell infiltration
TCR	T-cell receptor
TILs	Tumour-infiltrating lymphocytes
TMB	Tumour mutation burden
TME	Tumour microenvironment
TNM	Tumor, node, metastasis
TNT	Total neoadjuvant therapy
Tregs	regulatory T cells
TRG	Tumour regression grading
UICC	Union for International Cancer Control
W&W	watch and wait
XELOX	Xeloda and oxaliplatin
β2M	β2 microglobulin
